# Antiproliferative and photoprotective activities of the extracts and compounds from *Calea fruticosa*

**DOI:** 10.1590/1414-431X20209375

**Published:** 2020-07-17

**Authors:** T.M.Q. Seregheti, A.P.R. Pinto, M.da C. Gonçalves, A.dos S. Antunes, W.A.da S. Almeida, R.S. Machado, J.N. Silva, P.M.P. Ferreira, C. Pessoa, V.M.R. dos Santos, A.M. do Nascimento

**Affiliations:** 1 Departamento de Química, Instituto de Ciências Exatas e Biológicas, Universidade Federal de Ouro Preto, Campus Universitário Morro do Cruzeiro, Ouro Preto, MG, Brasil; 2 Departamento de Química, Programa de Pós-Graduação em Química, Instituto de Ciências Exatas e Biológicas, Universidade Federal de Ouro Preto, Campus Universitário Morro do Cruzeiro, Ouro Preto, MG, Brasil; 3 Departamento de Biofísica e Fisiologia, Laboratório de Cancerologia Experimental, Programas de Pós-Graduação em Ciências Farmacêuticas e Biotecnologia, Universidade Federal do Piauí, Teresina, PI, Brasil; 4 Departamento de Fisiologia e Farmacologia, Programa de Pós-Graduação em Farmacologia, Universidade Federal do Ceará, Fortaleza, CE, Brasil

**Keywords:** Flavone, Sesquiterpenic lactone, Flavonol, Glucosylated coumarin, Sunscreen preparation

## Abstract

In this paper, we complement our previous study on the antiproliferative activity of *Calea fruticosa* (Asteraceae) by isolating the compounds apigenin-4',7-dimethyl ether (1), budlein A (2), quercetin (3), and cichoriin (4) from the plant’s aerial parts. The antiproliferative activity of these compounds was evaluated by the 3-(4,5-dimethylthiazol-2-yl)-2,5-diphenyltetrazolium bromide (MTT) method against human tumor cell lines. Compound 3 displayed moderate antiproliferative activity in three cell lines (HCT-116, PC-3, and SF-295, with cell growth inhibition values of 72.97, 74.55, and 68.94%) and high antiproliferative activity (90.86%) in the HL-60 cell line. The *in vitro* sun protection factor (SPF) of the extracts and compound 4, with and without sunscreen, was determined by a spectrophotometric method. The ethanol extract exhibited the highest SPF (9.67) at a concentration of 0.100 mg/mL, while compound 4, isolated from this extract, showed a SPF of 13.79 at the same concentration. A relative increased efficacy of SPF was observed for the extracts and compound 4 when sunscreen was also used. Compound 4 has not been reported previously from any species within the genus *Calea*. Compounds 1–4 were obtained from this species for the first time.

## Introduction

Exposure to ultraviolet radiation has been implicated as a major causative agent and a risk factor for skin cancer (1). According to Oliveira Júnior et al. (2), the ultraviolet part of the electromagnetic spectrum is divided into three regions: ultraviolet A radiation (UVA - from 320 to 400 nm, it reaches the deeper layers of the epidermis and dermis and provokes the premature aging of the skin); ultraviolet B radiation (UVB - from 290 to 320 nm, it is not completely filtered out by the ozone layer and is responsible for sunburn damage), and ultraviolet C radiation (UVC - from 200 to 290 nm, it is filtered by the atmosphere before reaching the Earth's surface). Reducing the amount of UV electromagnetic radiation that reaches the skin with sunscreens may also reduce the risk of sunlight-induced skin cancer (3).

The use of plant extracts as an efficient strategy to protect against skin photoaging is growing. Many of these extracts have compounds with photoprotective or synergistic activity in association with sunscreens, in addition to their high antioxidant potential (4). Cancer is a major public health problem in the world (5). Many approaches in cancer management are often ineffective due to adverse reactions, drug resistance, or inadequate target specificity of single anticancer agents (6). Natural compounds from various sources offer a great opportunity for discovery of novel therapeutic candidates and plants have a long history of use in the treatment of cancer. Active constituents of *Catharanthus roseus* (Apocynaceae), *Angelica gigas* (Apiaceae), *Podophyllum peltatum* (Berberidaceae), *Taxus brevifolia* (Taxaceae), *Ochrosia elliptica* (Apocynaceae), and *Camptotheca acuminata* (Cornaceae) have been used in the treatment of advanced stages of various malignancies (7).

The great diversity of plants growing in Brazil offers interesting possibilities of finding novel photoprotective and anticancer compounds of natural origin. The genus *Calea* L., a member of the Asteraceae family, occurs in Mexico and Central and South America and contains approximately 110 species (8). Sesquiterpene lactones, especially furanoheliangolides, were isolated from these plants, but germacranolides, eudesmanolides, and guaianolides have also been found (9–12). Several species produce *p*-hydroxyacetophenone derivatives, chromanones, thymol derivatives, benzofurans, and other constituents (13–15). *Calea fruticosa* (Gardner) Urbatsch, Zlotsky & Pruski is a perennial herb with yellow flowers and is a synonym of *C. morii* H. Rob. (16). Other species belonging to the same genus are used in the treatment of stomach diseases (17–18).

Our research group has previously demonstrated the antiproliferative action of extracts from this species (19). However, we have not conducted studies on its chemical constituents, despite the importance of a more detailed phytochemical study. Considering the wide occurrence of the genus *Calea* L. in Brazil and the use of its different species in folk medicine, we now report the findings of the isolation and identification of *C. fruticosa* compounds with antiproliferative properties. We also aimed to investigate the photoprotective activity of these plant extracts in hexane, ethyl acetate, and ethanol, and of compound 4 alone and incorporated into sunscreen.

## Material and Methods

### General procedures

Silica gel 60 H (70–230 mesh; Merck No. 1.07736, Germany) and Polyamide CC6 (Macherey-Nagel, code 81561, Germany) were used in column chromatography. Preparative thin layer chromatography (TLC) was performed on silica gel GF_254_ (Merck No. 1.07730) (see Supplementary Figure S1 for details). ^1^H and ^13^C NMR spectra (1D experiments) were recorded on Bruker DRX 400 or Bruker DRX 500 spectrometers (400 or 500 MHz for ^1^H and 100 or 125 for ^13^C; Billerica, USA). DMSO-*d6* or pyridine-*d5* was used as solvent and TMS as an internal standard. Chemical shifts are reported in (δ) ppm and coupling constants (J values) in Hz. ^1^H and ^13^C NMR spectra (2D experiments, HSQC and HMBC) were performed using a Bruker DRX 400 spectrometer at 400 and 100 MHz, respectively. High-resolution electrospray ionization mass spectrometry (HR-ESI-MS) was performed on an UltrOTOF-Q Bruker-Daltonics instrument (Billerica) equipped with an ESI ion source and operating in positive and negative ion modes. Absorbance was measured using a UV/Visible spectrophotometer M51 (Bel Photonics, Brazil) equipped with 1 cm quartz cell.

### Plant material

Plants were collected in April 2012, in the state of Minas Gerais, Estrada do Calais, Parque Estadual do Itacolomi in the municipality of Ouro Preto, Brazil, and were identified by comparison with other voucher specimens previously identified and available in the herbarium, by Jorge Luiz da Silva. The voucher specimen (OUPR 26290) was deposited at the Herbarium José Badini, Universidade Federal de Ouro Preto (UFOP). The registries in SisGen (Sistema Nacional de Gestão do Patrimônio Genético e do Conhecimento Tradicional Associado – National System of Management of Genetic Heritage and Associated Traditional Knowledge) were performed (A59F25F) according to the Brazilian legislation on access to biodiversity (Federal Law No. 13,123/2015).

### Extraction and isolation

The air-dried and powdered aerial parts of *C. fruticosa* (46.2 g) were extracted successively with *n*-hexane, ethyl acetate, and ethanol by maceration at room temperature to give 0.9, 3.2, and 5.4 g of crude extracts. The ethyl acetate extract (3.2 g) was subjected to silica gel column chromatography with *n*-hexane, ethyl acetate, and ethanol, using mixtures to increase polarity. One hundred and thirty fractions were collected. Fractions 40–49 (147.0 mg) were combined and processed for preparative TLC (*n*-hexane/ethyl acetate 7:3) to yield 7.1 mg (0.015%) of compound 1. After preparative TLC (*n*-hexane/ethyl acetate 4:6), the combination of fractions 76–85 (364.0 mg) yielded 24.5 mg (0.053%) of compound 2. The ethanol extract (5.4 g) was chromatographed over reversed-phase polyamide using water and ethanol gradients, and ethanol and ethyl acetate gradients to yield a total of 70 fractions. Fractions 18–31 (103.0 mg) were rechromatographed on polyamide to give 9.6 mg (0.021%) of compound 3. When preparing the ethanol extract to be subjected to polyamide column, there was the formation of a precipitate (1.6 g), which was collected. Part of this precipitate (117.0 mg) was purified by recrystallization, using water as solvent. The white crystals produced (22.4 mg, 0.048%) are compound 4.

### Antiproliferative assay

The antiproliferative potential of the compounds isolated from *C. fruticosa* was evaluated by the MTT assay (20), against four human tumor cell lines: HCT-116 (colorectal carcinoma), HL-60 (promyelocytic leukemia), PC-3 (prostate cancer), and SF-295 (glioblastoma), all obtained from the National Cancer Institute (USA), according to a procedure described in literature (21). Tumor cell growth was quantified by the ability of living cells to reduce the yellow dye 3-(4,5-dimethyl-2-thiazolyl)-2,5-diphenyl-2H-tetrazoliumbromide (MTT) and form an insoluble purple formazan product. Cells were plated in 96-well plates [0.1×10^6^ cells/mL (PC-3 and SF-295), 0.3×10^6^ cells/mL (HL-60), and 0.7×10^5^ cells/mL (HCT-116)] and the isolated compounds were added to each well (50 μg/mL). Doxorubicin (0.3 μg/mL, Sigma Aldrich, Canada) was used as positive control.

### Photoprotective assay

#### In vitro determination of the sun protection factor (SPF)

The *in vitro* SPF of the extracts and compound 4 from *C. fruticosa*, with and without sunscreen, was determined by a spectrophotometric method developed by Mansur et al. (22). Dry extracts and compound 4 were diluted to give the following concentrations of 0.020 (only for compound 4), 0.030, 0.050, 0.070, and 0.100 mg/mL. The absorbance of the samples was measured in the UVB wavelength range (290–320 nm). The results of the SPF are reported as the arithmetic mean of three measurements.

#### Incorporation of the extracts and compound 4 into the sunscreen

The sunscreen UVA-UVB 5% gel hydrosoluble Pemulen TR-1^®^ was purchased from BioFarma (Brazil). The UVA/UVB sunscreen is a commercially available combination of 2-phenylbenzimidazole-5-sulfonic acid (Eusolex 232^®^) and 2-hydroxy-4 methoxybenzophenone. The sunscreen UVA-UVB 5% Pemulen TR-1 gel (1 g) and 1 mL of solution (1 mg/mL) of each one of the crude extracts and compound 4 were separately added to a 100-mL beaker. Each mixture was maintained under stirring for 30 min at room temperature and, after that, the sample was stored (23).

#### In vitro determination of the SPF after incorporation of the extracts or compound 4 into the sunscreen

Portions of 1 g of the sunscreen UVA-UVB 5% Pemulen TR-1 gel (incorporated with the extracts or compound 4, and the sunscreen UVA-UVB 5% Pemulen TR-1 gel alone) were weighed. Subsequently, dilutions of this material in 70% ethanol were performed in triplicate to obtain a concentration of 0.2 μL/mL (22,23).

### Statistical analysis

Cell proliferation (*in vitro* antiproliferative assays) was determined by nonlinear regression using the GraphPad Prism 6.0 Software (USA). The analysis of the SPF was performed in triplicate and the results are reported as means±SD.

## Results

### Structural identification of compounds isolated from ***C. fruticosa***

**Figure 1:**
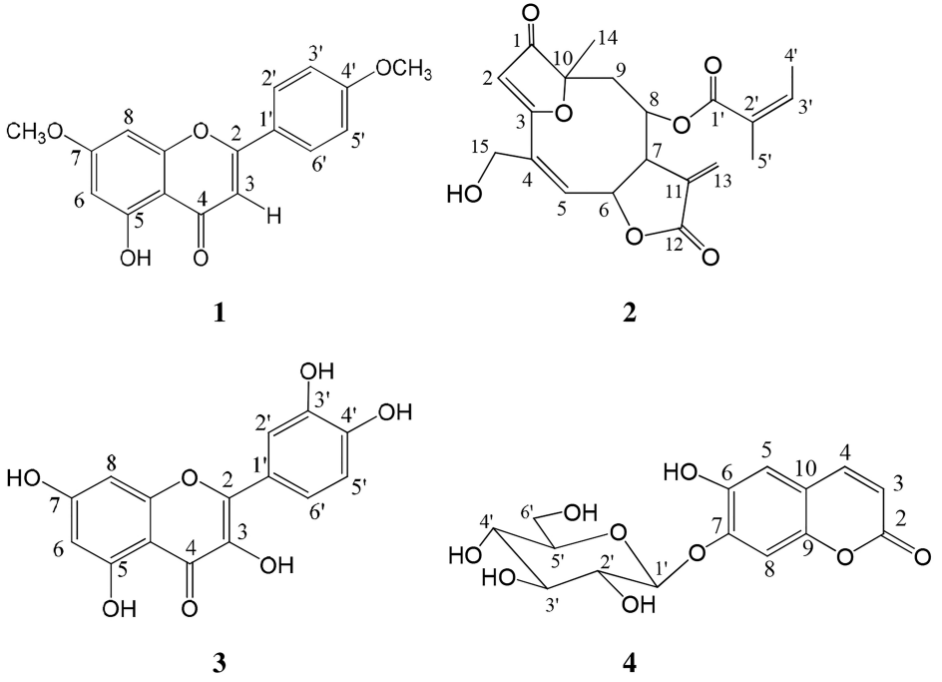
Structural representations for apigenin-4',7-dimethyl ether (compound 1), budlein A (compound 2), quercetin (compound 3), and cichoriin (compound 4) isolated from the aerial parts of *C. fruticosa*.

Compounds 1–4 (Figure 1, structural representations of the compounds) were isolated as described in the Materials and Methods section. Compounds 1 and 2 were isolated from the ethyl acetate extract, whereas compounds 3 and 4 were isolated from the ethanol extract of the aerial parts of *C. fruticosa*. The phytochemical screening of the crude extracts was previously described by our research group (19). Chemical tests were carried out on the crude extracts to identify alkaloids, flavonoids, saponins, tannins, and terpenoids. The phytochemical screening of *C. fruticosa* ethyl acetate extract showed the presence of flavonoids and terpenoids. A flavone (1) and a sesquiterpenic lactone (2) were isolated from this extract, corroborating the results of the screening. The ethanol extract showed positive results for the presence of flavonoids, saponins, and tannins. A flavonol (3) and a glucosylated coumarin (4) were isolated from this extract.

Compound 1 (7.1 mg) was isolated as a yellow gum and displayed a characteristic ^1^H NMR spectrum, with the same substitution pattern of apigenin rings: A (5,7-disubstituted), with two signals at δ 6.40 (d, 1H, J=2.3 Hz, H-6) and 6.82 (d, 1H, J=2.3 Hz, H-8), and B (4-monosubstituted), with two signals at δ 7.14 (d, 2H, J=8.8 Hz, H-3'/5') and 8.09 (d, 2H, J=8.8 Hz, H-2'/6'). ^1^H NMR spectrum also showed the presence of two methyl ether groups replaced in the positions 7 and 4 and a 5-OH signal. Compared with data from the literature (24), the NMR data of compound 1 allowed its assignment as 5-hydroxy-4',7-dimethoxyflavone, also known as apigenin-4',7-dimethyl ether, genkwanin-4'-methyl ether, or acacetin-7-*O*-methyl ether (7-*O*-methylacacetin). This flavone is being isolated from *C. fruticosa* for the first time, but it has been isolated from other species of the same genus, including *C. zacatechichi* (9,18), *C. divaricata* (25), *C*. *septuplinervia* (11), and others.

Compound 2 was isolated as a white gum. The molecular formula C_20_H_22_O_7_ was confirmed for compound 2 (24.5 mg) by the HRMS (ESI), in which a pseudo-molecular ion peak at *m/z* 397.1068 [M+Na]^+^ was observed in the positive ion mode. The ^1^H NMR spectrum of compound 2 showed a pair of low-field doublets at *δ* 6.15 and 5.98, characteristic of a γ-lactone conjugated with an exocyclic methylene group; two singlets signals centered at *δ* 5.24 and 5.11 (H-6 and H-8); a singlet at *δ* 4.18 (2H) assigned to the –CH_2_–O– hydrogens of an allylic primary alcohol; a signal corresponding to H-7 (δ 3.71); signals for the vinyl methyl groups (*δ* 1.82 and 1.72) of the angelate ester moiety; a singlet at *δ* 1.36 for the C-14 methyl group. Compound 2 was confirmed as a sesquiterpene lactone budlein A by comparison between its ^1^H and ^13^C NMR spectra and those published in the literature (26). This compound is also being isolated for the first time from *C. fruticosa* in this study, but it has been found in *C. zacatechichi*(9), *C. divaricata*(25), *C. hispida*(27), *C. hymenolepis*(28), and *C. villosa*(29).

Compound 3 was isolated as a yellow amorphous powder. The ^1^H NMR data of compound 3 (9.6 mg) showed two signals at *δ* 6.18 (d, 1H, J=2.0 Hz, H-6) and 6.40 (d, 1H, J=2.0 Hz, H-8) that were consistent with the *meta* hydrogens H-6 and H-8 on A-ring, and an ABX system at *δ* 7.67 (d, 1H, J=2.0 Hz, H-2'), 7.53 (dd, 1H, J=2.0 Hz, 8.4 Hz, H-6'), and 6.88 (d, 1H, J=8.4 Hz, H-5') that corresponded to the catechol hydrogens on B-ring. Thus, compound 3 was identified as 3,5,7,3',4'-pentahydroxyflavone, also known as quercetin. ^1^H and ^13^C data were consistent with those reported for this compound in the literature (30). This compound has already been isolated from *C. platylepis*(31), although its isolation from the species under study has not been published.

Compound 4 was isolated as a white amorphous powder. The molecular formula C_15_H_16_O_9_ was determined for compound 4 (22.4 mg) based on the HRMS (ESI), in which a pseudo-molecular ion at *m/z* 339.0830 [M−H]^−^ was observed in the negative ion mode. In the ^1^H NMR spectrum, doublets at *δ* 6.34 (J=9.6 Hz, H-3) and 7.63 (J=9.6 Hz, H-4) have been observed, which are characteristic of benzo-α-pyrone derivatives. This spectrum also showed two singlets at δ 7.28 (H-5) and 7.55 (H-8). The presence of one β-glucosyl moiety, with characteristic signals at δ 5.82 (d, J=7.8 Hz, H-1') of anomeric hydrogen and 4.16−4.57 (H-2' to H-6'), was confirmed. The glucose moiety was replaced at 7-O position based on the HMBC spectrum. HMBC correlations were observed between H-1' (anomeric hydrogen) of the glucose and carbon resonance at *δ* 150.3 (C-7). Compound 4 was identified as 6,7-dihydroxycoumarin-7-O-β-glucopyranoside (32), also known as esculetin-7-O-β-glucopyranoside or cichoriin. To our knowledge, this compound is being reported for the first time in this species and genus of plant.

### Spectroscopic data of compounds 1, 2, 3, and 4

Apigenin-4',7-dimethyl ether (5-hydroxy-4',7-dimethoxyflavone, 1): ^1^H NMR (400 MHz, DMSO-*d6*) *δ* 3.87 (s, 3H, OCH_3_), 3.88 (s, 3H, OCH_3_), 6.40 (d, 1H, *J* 2.3, H-6), 6.82 (d, 1H, *J* 2.3, H-8), 6.96 (s, 1H, H-3), 7.14 (d, 2H, *J* 8.8, H-3'/5'), 8.09 (d, 2H *J* 8.8, H-2'/6'), 12.94 (s, 1H, OH-5) (Supplementary Figure S1).

Budlein A (2): ^1^H NMR (500 MHz, DMSO-*d6*) *δ* 1.36 (s, 3H, H-14). 1.72 (s, 3H, H-5'), 1.82 (dq, 3H, *J* 7.3, 1.3, H-4'), 2.33 (dd, 1H, *J* 15.3, 5.4 H-9β), 2.55 (dd, 1H, *J* 15.3, 5.4, H-9α), 3.71 (s, 1H, H-7), 4.18 (s, 2H, H-15), 5.24 (s, 1H, H-6), 5.11 (s, 1H, H-8), 5.98 (d, 1H, *J* 2.4, H-13a), 5.85 (s, 1H, H-2), 6.09 (m, 2H, H-5/H-3'), 6.15 (d, 1H, *J* 2.6, H-13b); ^13^C NMR (125 MHz, DMSO-*d6*) *δ* 15.3 (C-4'), 19.6 (C-5'), 20.9 (C-14), 41.1 (C-9), 47.5 (C-7), 60.7 (C-15), 74.9 (C-8), 75.0 (C-6), 87.3 (C-10), 104.3 (C-2), 123.8 (C-13), 126.5 (C-2'), 132.1 (C-5), 136.9 (C-4), 138.8 (C-11), 139.8 (C-3'), 165.4 (C-1'), 168.5 (C-12), 183.0 (C-3), 205.1 (C-1); HRMS (ESI) *m/z*, observed: 397.1068; C_20_H_22_O_7_ [M+Na]^+^ required: 397.3762 (Supplementary Figures S2, S3, S4, S5).

Quercetin (3,5,7,3',4'-pentahydroxyflavone, 3): ^1^H NMR (400 MHz, DMSO-*d6*) *δ* 6.18 (d, 1H, *J* 2.0, H-6), 6.40 (d, 1H, *J* 2.0, H-8), 6.88 (d, 1H, *J* 8.4, H-5'), 7.53 (dd, 1H, *J* 8.4, 2.0, H-6'), 7.67 (d, 1H, *J* 2.0, H-2'); ^13^C NMR (100 MHz, DMSO-*d6*) *δ* 94.4 (C-8), 99.2 (C-6), 104.0 (C-10), 116.1 (C-2'), 116.6 (C-5'), 121.0 (C-6'), 123.0 (C-1'), 136.8 (C-3), 146.1 (C-3'), 147.8 (C-4'), 148.8 (C-2), 157.1 (C-9), 161.8 (C-5), 164.9 (C-7), 176.9 (C-4) (Supplementary Figures S6, S7).

Cichoriin (6,7-dihydroxycoumarin-7-O-β-glucopyranoside, 4): ^1^H NMR (500 MHz, pyridine-*d5*) *δ* 4.18 (m, 1H, H-3'), 4.28 (m, 1H, H-2'), 4.34 (m, 1H, H-4'), 4.39 (m, 1H, H-5'), 4.43-4.57 (m, 2H, H-6'), 5.82 (d, 1H, *J* 7.8, H-1'), 6.34 (d, 1H, *J* 9.6, H-3), 7.28 (s, 1H, H-5), 7.55 (s, H1, H-8), 7.63 (d, 1H, *J* 9.6, H-4); ^13^C NMR (125 MHz, pyridine-*d5*) *δ* 62.2 (C-6'), 71.1 (C-4'), 74.7 (C-2'), 78.4 (C-5'), 79.2 (C-3'), 102.8 (C-1'), 105.0 (C-8), 113.9 (C-5), 114.1 (C-10), 114.3 (C-3), 143.7 (C-4), 145.8 (C-6), 148.8 (C-9), 150.3 (C-7), 161.2 (C-2); HRMS (ESI) *m/z*, observed: 339.0830; C_15_H_16_O_9_ [M−H]^−^ required: 339.2757) (Supplementary Figures S8, S9, S10, S11).

### Antiproliferative assay

Table 1 summarizes the results of the assays on the cytotoxicity of the compounds isolated from *C. fruticosa* extracts, with the exception of compound 1 (5-hydroxy-4',7-dimethoxyflavone), which was isolated from the ethyl acetate extract because it has not presented itself in a pure form.

**Table 1:** *In vitro* antiproliferative activity of compounds 2–4 isolated from the aerial parts of *C. fruticosa*.

Samples	Cell growth inhibition (%)			
	HCT-116	HL-60	PC-3	SF-295
Compound 2	4.59±2.48	7.54±00.0	7.68±3.65	2.71±2.34
Compound 3	72.97±1.71	90.86±0.77	74.55±0.80	68.94±1.11
Compound 4	4.14±00.0	11.65±3.46	16.01±2.37	2.76±2.89
Doxorubicin (positive control)	96.80±4.60	100.00±0.70	95.6±5.98	83.60±3.00

Data are reported as means±SD based on two independent experiments for colorectal carcinoma (HCT-116), promyelocytic leukemia (HL-60), prostate (PC-3), and glioblastoma (SF-295) human cancer cells. Doxorubicin (0.3 μg/mL) was used as positive control. High antiproliferative activity: >75%; moderate activity: 50 to 75%; low activity: <50%. Compound 2: budlein A; Compound 3: quercetin; Compound 4: cichoriin. No antiproliferative activity was determined for Compound 1 as it did not present itself in a pure form.

Compound 2 showed a low antiproliferative activity in three human tumor cell lines (HCT-116, PC-3, and SF-295) and no antiproliferative activity in the HL-60 cell line. Compound 3 showed a moderate antiproliferative activity in three cell lines (HCT-116, PC-3, and SF-295, with values of 72.97, 74.55, and 68.94%, respectively) and a high activity (90.86%) in HL-60 cells. Compound 4 showed low antiproliferative activity results in all human tumor lines used.

### Photoprotective assay

The results of the *in vitro* determination of the SPF values of the crude extracts are shown in Table 2. It can be observed that the SPF values found for the hexane and ethyl acetate extracts were lower than 6 in all concentrations tested (0.030, 0.050, 0.070, and 0.100 mg/mL). In this way, these extracts do not exhibit photoprotective activity, since according to the Brazilian resolution RDC 30, of June 1st, 2012 (33), the SPF must be at least 6 for a product to be considered as a true sunscreen. Only the ethanol extract showed higher SPF, with values of 6.9625 and 9.6665 at concentrations of 0.070 and 0.100 mg/mL, respectively. The SPF values of the extracts tested were concentration-dependent, and the increase in their concentration resulted in an increment in the SPF. The data for the positive control were taken from another study of our research group (34).

**Table 2:** Sun protection factor (SPF) of extracts from the aerial parts of *C. fruticosa* and positive control (UVA-UVB 5% Pemulen TR-1 gel) at different concentrations.

Concentration	*n*-Hexane extract	Ethyl acetate extract	Ethanol extract	Positive control (34)
0.030 mg/mL	0.0451±0.0006	1.6005±0.0184	2.7784±0.0307	5.42±1.67
0.050 mg/mL	0.1449±0.0017	2.6837±0.0309	4.3449±0.0481	10.36±3.19
0.070 mg/mL	0.1964±0.0024	3.6930±0.0424	6.9625±0.0770	13.86±4.27
0.100 mg/mL	0.1429±0.0018	5.2550±0.0604	9.6665±0.1069	19.75±6.07

Data are reported as means±SD. The data for the positive control was taken from reference 34.

Coumarin (4) was also isolated from the ethanol extract and the results of the *in vitro* determination of the SPF values for this compound are shown in Table 3. It can be observed that the SPF values found for compound 4 are higher than those found for the ethanol extract. Compound 4 was identified as 6,7-dihydroxycoumarin-7-O-β-glucopyranoside, also known as esculetin-7-O-β-glucopyranoside, and the photoprotective properties of esculetin (aglycone without the glycoside in position 7) were reported by Lee et al. (35). Esculetin exhibits a good inhibitory activity on the UV-induced expression of metalloproteinase 1 (MMP-1), also known as collagenase-1, an enzyme responsible for the degradation of collagen. Based on this result, esculetin was considered to be potentially useful as an ingredient in cosmetics for prevention of skin photoaging.

**Table 3:** Sun protection factor (SPF) of compound 4 and positive control (UVA-UVB 5% Pemulen TR-1 gel) at different concentrations.

Concentration	Compound 4	Positive Control (34)
0.020 mg/mL	6.4197±0.6862	4.16±1.28
0.030 mg/mL	7.4602±0.7931	5.42±1.67
0.050 mg/mL	10.0737±1.0698	10.36±3.19
0.070 mg/mL	12.6292±1.3410	13.86±4.27
0.100 mg/mL	13.7900±4.1400	19.75±6.07

Data are reported as means±SD. Compound 4: cichoriin. No photoprotective activity was determined for Compound 3, because it did not have enough mass, 10 mg. The data for the positive control was taken from reference 34.

The results of the *in vitro* determination of SPF values of the crude extracts and compound 4 incorporated into the sunscreen are shown in Table 4. All the extracts caused an intensification of the SPF through a synergistic action with the sunscreen. The ethanol extract and ethyl acetate extract presented the highest SPF when incorporated into the sunscreen and the hexane extract exhibited the lowest SPF (very close to the value of sunscreen). The SPF of the ethanol extract with the sunscreen was higher than that of compound 4 with the sunscreen, which may be due to the presence of quercetin (3).

**Table 4:** Sun protection factor (SPF) of the UVA-UVB 5% Pemulen TR-1 gel sunscreen, and extracts and compound 4 incorporated into the sunscreen.

Formulation (0.20 μL/mL)	SPF
Sunscreen UVA-UVB 5% gel	10.22±3.16
*n*-Hexane extract with sunscreen UVA-UVB 5% gel	10.81±3.33
Ethyl acetate extract with sunscreen UVA-UVB 5% gel	18.59±5.71
Ethanol extract with sunscreen UVA-UVB 5% gel	19.12±5.80
Compound 4 with sunscreen UVA-UVB 5% gel	14.46±4.48

Data are reported as means±SD. Compound 4: cichoriin.

## Discussion

This paper represents the first study on the secondary metabolites of *C. fruticosa*. We have previously described the cytotoxic action of these extracts (19). The hexane crude extract showed a high antiproliferative activity (95.3%) on OVCAR-8 (ovarian) human tumor cell line, moderate activity (72.7%) on HCT-116 (colorectal carcinoma), and low activity (45.2%) on SF-295 (glioblastoma). The ethyl acetate extract exhibited a strong antiproliferative effect on HCT-116, OVCAR-8, and SF-295, with values of 99.4, 96.7, and 96.2%, respectively. The ethanol extract showed a high antiproliferative activity on HCT-116 and OVCAR-8 (95.7 and 95.6%) and moderate activity on SF-295 (61.2%). This study evaluated the antiproliferative action of 3 major compounds: budlein A (2), quercetin (3), and cichoriin (4), isolated from active *C. fruticosa* extracts, also using the *in vitro* MTT method. The results are reported in terms of % cell growth inhibition. Compounds 2 and 4 showed no antiproliferative activity, while compound 3 showed a moderate to high antiproliferative action. These results indicated that quercetin (3) was, at least in part, responsible for the high and moderate antiproliferative activity observed for the ethanol extract in HCT-116 and SF-295 cell lines, respectively (19). Molecular targets of quercetin (3) for prevention of cancer have already been described in the literature (36). Quercetin governs numerous intracellular targets, including proteins involved in apoptosis, cell cycle, detoxification, antioxidant replication, and angiogenesis. The available synergistic studies strongly suggest the use of quercetin as a chemoprevention drug (37). There may also be other active compounds at low concentrations, responsible for the promising antiproliferative effect of the ethanol extract and that have not been identified. Therefore, results of this study highlight *C. fruticosa* as potential source in the search for anticancerous agents.

This study also evaluated the photoprotective properties of *C. fruticosa* extracts and cichoriin (4), using the spectrophotometric method developed by Mansur et al. (22). The ethanol extract showed the highest SPF (9.6665±0.1069) at a concentration of 0.100 mg/mL. The photoprotective activity of the ethanol extract can be attributed to the avonol quercetin (3) isolated from this extract. This compound is one of the most potent antioxidants (38), and its photoprotective properties could contribute to its antioxidant action (39). The SPF of quercetin using the method developed by Mansur et al. (22) was 5.46, 8.71, 12.28, 15.19, and 18.36 at concentrations of 0.010, 0.015, 0.020, 0.025, and 0.030 mg/mL (40). Compound 4, isolated from this extract, showed an SPF of 13.7900±4.1400 at a concentration of 0.100 mg/mL, which indicated that this particular compound might be one of the main constituents responsible for the photoprotective action of the ethanol extract, along with quercetin (3).

Among the active substances present in plants that can be used to provide broader skin photoprotection when added to formulations are antioxidants such as vitamin C and E, tannins, alkaloids, and flavonoids (4). The ethanol extract showed positive results for the presence of flavonoids, saponins, and tannins in the phytochemical screening (19). Compounds 3 and 4 were isolated from this extract and, together with those detected in the phytochemical screening, could substantially affect photoprotection synergistically.

The present study demonstrated the importance and interest of using extracts and substances isolated from *C. fruticosa* in sunscreen preparations with the incorporation of an UVA-UVB 5% gel. A relative SPF increase was observed for these preparations, leading to increased protection against the sun's harmful rays. The ethanol extract exhibited higher sun protection. Our results contribute to the better understanding of Brazil's plant biodiversity and indicate that these natural sources may become important sources for therapeutic and photoprotective agents.

## Supplementary Material

Click here to view [pdf].
